# Cancer burden and status of cancer control measures in fragile states: a comparative analysis of 31 countries

**DOI:** 10.1016/S2214-109X(22)00331-X

**Published:** 2022-09-13

**Authors:** Isabel Mosquera, Andre Ilbawi, Richard Muwonge, Partha Basu, Andre L Carvalho

**Affiliations:** a Early Detection, Prevention, and Infections Branch, International Agency for Research on Cancer, Lyon, France; b Department of Non-Communicable Diseases, WHO, Geneva, Switzerland

## Abstract

**Background:**

Information on cancer statistics and cancer control policies is limited in fragile states. This paper describes the cancer burden and status of cancer control measures in these countries.

**Methods:**

In this comparative analysis, fragile states presenting with a Fragile States Index (FSI) score of 90·0 or more (alert for fragility) for at least 10 years during the 2006–20 period were selected. States with fewer than 10 years of data were selected if they were in alert for fragility during all years. Information on cancer burden, prevalence of cancer risk factors, population-attributable fraction, and on political commitment, health financing, and health system capacity was collected. Cancer incidence and mortality was calculated on the basis of data from population-based cancer registries, estimated with modelling that used mortality-to-incidence ratios and incidence-to-mortality ratios derived from cancer registries in neighbouring countries, or average of rates in selected neighbouring countries. For statistical comparison, fragile states were grouped according to the annual percent change (APC) of the FSI, with group 1 showing an increasing fragility trend (APC 0·2% or higher), group 2 a relatively stable fragility trend (APC between 0·2% and –0·2%), and group 3 a decreasing fragility trend (APC of –0·2% or lower).

**Findings:**

Overall, the estimated cancer burden in the 31 selected fragile states was lower than worldwide rates, except for cervical and prostate cancer. Cancer cases were attributed to infections (22·40% in group 1, 21·20% in group 2, and 18·80% in group 3) at a higher proportion in fragile states than globally (13·0%). Group 1 and 2 showed a significantly higher exposure to household air pollution (97·70% in group 1 and 94·90% in group 2), whereas current tobacco use in men increased from group 1 to group 3, with lung cancer incidence and mortality being higher in group 3. However, 25 countries had implemented only one or no MPOWER measures for tobacco control. Countries showed an out-of-pocket expenditure of 48·72% in group 1, 42·68% in group 2, and 51·07% in group 3, and only half of the countries had an updated cancer control plan or cancer management guidelines.

**Interpretation:**

Fragile states have started the epidemiological transition but are still not implementing enough cancer control measures. There is a need to develop reliable cancer control plans and guidelines, and to create financial mechanisms for implementation.

**Funding:**

None.

**Translations:**

For the Arabic and French translations of the abstract see Supplementary Materials section.

## Introduction

Fragility of a state is a complex and evolving concept, widely used among donor organisations and agencies supporting development and peacebuilding. Fragility has been characterised by the Organisation for Economic Co-operation and Development as a situation in which state, system, and communities are exposed to multidimensional risks, but have limited capacity to manage, absorb, and mitigate them.^1^ The World Bank defines fragile countries as those that are not in medium-intensity or high-intensity conflict (such countries are considered as being beyond fragile) and fulfil at least one of the following criteria: the Country Policy and Institutional Assessment score (used to assess conduciveness to poverty reduction, sustainable growth, and effective use of development assistance) is lower than 3·0; the presence of a UN peacekeeping operation; or having 2000 or more people per 100 000 population migrating across borders.^2^

Fund For Peace developed the Failed States Index to measure fragility, and subsequently replaced it with the Fragile States Index (FSI), aiming to better understand and measure conflict drivers and dynamics in complex environments. On the basis of the Conflict Assessment System Tool framework, the FSI score considers 12 indicators classified into four categories, comprising cohesion, economic, political, and social and cross-cutting categories. Cohesion indicators measure security apparatus, factionalised elites, and group grievance. Economic indicators refer to economic decline, uneven economic development, and human flight and brain drain. Political indicators assess state legitimacy, public services, and human rights and rule of law. Lastly, social and crosscutting indicators examine demographic pressures, refugees, internally displaced people, and external intervention.^3^

**Research in context**

**Evidence before this study**

We searched PubMed in January, 2020, without language or time period restrictions using the following combination of words in titles or abstracts: “cancer”, “incidence”, “mortality”, “survival”, “control”, “screening”, and “fragile states”. This search produced no results; therefore, “fragile states” was substituted by countries with a Fragile State Index showing an alert for fragility in 2019 (“Yemen”, “Somalia”, “Congo Democratic Republic”, “Central African Republic”, “Chad”, “Sudan”, “Afghanistan”, “Zimbabwe”, “Guinea”, “Haiti”, “Iraq”, “Nigeria”, “Burundi”, “Cameroon”, “Eritrea”, “Niger”, “Guinea Bissau”, “Uganda”, “Myanmar”, “Pakistan”, “Ethiopia”, “Kenya”, “Côte d’Ivoire” and “Liberia”). Although we identified several studies for individual countries, there was a scarcity of data for fragile states as a whole, and an absence of a comprehensive analysis or benchmark comparison among them. When available, limited analyses were mainly focused on cancer burden in sub-Saharan Africa.

**Added value of this study**

To the best of our knowledge, this study is the first providing a comprehensive overview of cancer burden and cancer control measures in the fragile states. Overall, we could observe that an epidemiological transition from communicable to non-communicable diseases is happening among fragile states. Group 3 (countries with a decreasing fragility trend) begins to present a spectrum of exposure to cancer risk factors towards those common in more developed countries, and a rise in cancer cases related to those risk factors. At the same time, we observed that fragile states are not getting ready for the expected rise in cancer burden, presenting a lack of cancer control measures related to cancer prevention, health system capacity, and formulating response. There is a clear need to invest in cancer control planning and implementation.

**Implications of all the available evidence**

The findings will inform policy makers on the priorities fragile states should focus on. Many of these countries are undergoing demographical ageing and an epidemiological transition from communicable to non-communicable diseases. This transition will entail an increase in cancer burden and a shift in the predominant cancer types linked to a change in life expectancy and cancer risk-factor profile. There is a strong need for a better information system and reliable population statistics to better inform policy makers. Although most cancer cases associated with infections can be prevented, they represent 20% of cancer cases in fragile states, higher than the worldwide proportion of 13%. Despite a high burden of cervical cancer, less than 20% of countries have been able to introduce human papillomavirus vaccination in their national immunisation programme, even though more than 75% of the countries are eligible for Gavi, the Vaccine Alliance support, while coverage of cervical cancer screening is either low or unknown. The data collected suggest that countries are not ready for the expected rise in cancer burden and need to invest in cancer control planning. Fragile states have limited capacity to manage, absorb, and mitigate this trend; thus, they need a reliable plan linked to a financing mechanism for implementation and systematic monitoring and evaluation of the implementation process and sustainability of cancer control activities.

Studies assessing health in fragile states mainly focus on countries in conflict and with infectious disease outbreaks,^4^ whereas non-communicable diseases are frequently overlooked.^5^ Most fragile states are in Africa and Asia, the continents that account for 55·0% of new cancer cases and 65·5% of cancer deaths estimated in 2020.^6^ Data related to the burden or trend of cancer in fragile states is scarce, even though many of them are undergoing the social and economic transitions that augment risk of disease in the population. Moreover, evidence on the quality and comprehensiveness of cancer control planning, financial feasibility, and status of programme implementation in fragile states is scarce. Thus, the objective of this study is to describe the cancer burden and status of cancer control measures in fragile states.

## Methods

### Selection of fragile states

In this comparative analysis the selection of fragile states was based on the FSI score,^3^ which can range from 0 to 120. This score classifies 178 countries as sustainable (score <30·0), moderately fragile (score 30·0–59·9), in warning of fragility (score 60·0–89·9), or in alert for fragility (score ≥90·0). Countries were selected for this study if their FSI showed an alert for fragility for at least 10 years during a 15-year period (since inception of the score in 2006 up to 2020) or, for countries with less than 10 years of data, if they were in alert for fragility during all years.

### Data sources

Information collected for each country included cancer burden, prevalence of cancer risk factors, and population-attributable fraction (PAF) for common risk factors. We also recorded the status of political commitment, health financing, and health system capacity measured by health workforce and infrastructure available to deliver cancer diagnostic and treatment services. Data on incidence and mortality for all cancers and selected cancer sites (breast, cervix, colon, lung, prostate, and rectum) was calculated on the basis of data from population-based cancer registries from Cameroon, Congo (Brazzaville), Côte d’Ivoire, Ethiopia, Guinea, Kenya, Nepal, Niger, Nigeria, Pakistan, Sierra Leone, Sudan, Uganda, and Zimbabwe. For other countries they were estimated by modelling using mortality-to-incidence (M:I) ratios and incidence-to-mortality ratios derived from cancer registries in neighbouring countries, or as an average of rates in selected neighbouring countries.^7^ Incidence and mortality were expressed as age-standardised rates (ASRs; standardised to the world population) per 100 000 person-years.^7^ We reported disability-adjusted life-years (DALYs) caused by cancer.^8^ The M:I ratio, which is considered a surrogate for survival, was calculated for all cancers and selected cancer sites.

For cancer control planning we included information on non-communicable diseases integrated plans, cancer plans, and cancer management guidelines.^9^ As a measure of external evaluation, we included information on the number of countries having had an integrated mission of Programme of Action for Cancer Therapy (imPACT) review mission. This is a tool used by the International Atomic Energy Agency, with WHO and the International Agency for Research on Cancer (IARC), to do a situational analysis and provide recommendations for the cancer control of the country.^10^

We assessed the implementation of measures on monitor tobacco use and prevention policies, protect people from tobacco smoke, offer help to quit smoking, warn against the dangers of tobacco, enforce bans on tobacco advertising, promotion, and sponsorship, and raise taxes on tobacco (MPOWER). MPOWER was considered to be fully implemented if the following conditions were met: there was recent, representative, and periodic data for both adults and younger people (aged 13 years and older); all public places were completely smoke free (or at least 90% of the population covered by complete subnational smoke-free legislation); there was a national quit line, and both nicotine-replacement therapies and some cessation services were cost covered; there were large warnings with all appropriate characteristics on cigarette packages and national anti-tobacco campaigns were done with at least seven appropriate characteristics, including airing on television, radio, or both; there was a ban on all forms of direct and indirect advertising (or at least 90% of the population covered by subnational legislation completely banning tobacco advertising, promotion, and sponsorship); and 75% of the retail price or higher was tax.^11^

The information for each country under consideration was collected mainly from the Global Cancer Observatory of the IARC,^7^ and the WHO country capacity survey^9^ and cancer country profiles (sources are provided in the appendix 3 p 1).^7^, ^8^, ^9^, ^10^, ^11^, ^12^, ^13^, ^14^, ^15^, ^16^, ^17^, ^18^, ^19^, ^20^, ^21^, ^22^, ^23^, ^24^, ^25^

### Statistical analysis

The annual percent change (APC) of the FSI was calculated considering zero joinpoints, and starting from the first year (2007 for the Congo [Brazzaville] and Timor Leste, 2012 for South Sudan, and 2006 for the other countries) up to the last year that FSI was available (2020). Countries were then grouped in terciles of APC of FSI, and kept in the same group those with the same APC of FSI. Group 1 included countries with an APC of 0·2% or higher (countries with an increasing fragility trend), group 2 those with an APC between 0·2% and –0·2% (countries with a relatively stable fragility trend), and group 3 those with an APC of –0·2% or lower (countries with a decreasing fragility trend). For continuous variables, we calculated median and quartiles. We compared the overall difference among the three groups using a Kruskal-Wallis test and in between two groups using a Mann-Whitney's test. A p value lower than 0·05 was used to infer significant difference. For qualitative variables, we calculated the number of countries in each group with a particular result. Worldwide rates have been included to facilitate comparison. The statistical software that we used were Joinpoint regression programme 4.8.0.1 and SPSS Statistics 27.

### Role of the funding source

This study had no funder.

## Results

Globally, there were 31 countries showing alert for fragility for the period considered and these were mostly part of Africa and Asia (figure A). The countries in group 1, that is, those with increasing fragility trend, included Yemen, Eritrea, Central African Republic, Guinea Bissau, Cameroon, Niger, Nigeria, Burundi, and South Sudan. The countries in group 2, with an APC between 0·2% and –0·2%, included Afghanistan, Democratic Republic of the Congo, Ethiopia, Somalia, Kenya, Congo (Brazzaville), Uganda, Haiti, Chad, Guinea, and Liberia. The countries in group 3, with with a decreasing fragility trend, included Sudan, Myanmar, North Korea, Pakistan, Sierra Leone, Iraq, Nepal, Bangladesh, Zimbabwe, Timor-Leste, and Côte d’Ivoire (figure B).

**Figure:**
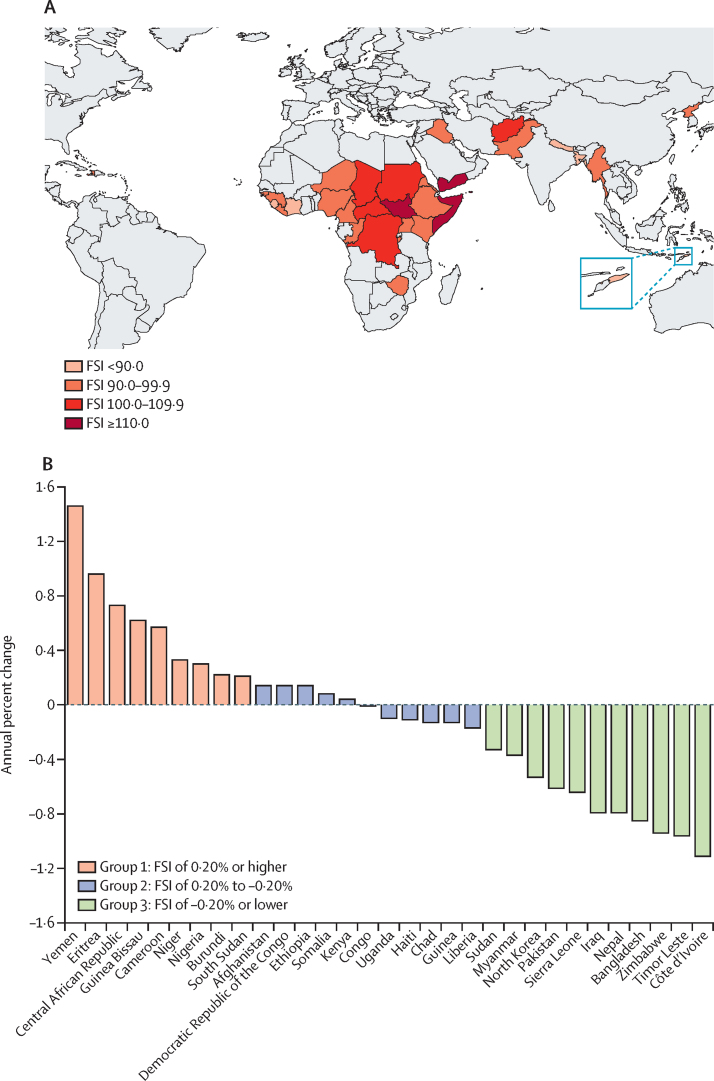
Annual percent change in FSI trend and map of FSI in selected countries (A) Countries by overall annual percent change in FSI trend. (B) 2020 FSI of assessed countries. FSI=Fragile States Index.

Median ASR of cancer incidence ranged from 100·80 per 100 000 person-years (25th percentile to 75th percentile 95·85–119·00) among group 1 countries to 116·60 per 100 000 person-years (102·90–137·90) among group 2 countries, without any significant difference across the groups (p=0·33; table 1). We observed a significantly higher prevalence of all cancers in group 3 than in group 1 (p=0·0027) and group 2 (p=0·020). Further analysis for selected tumour sites showed that prostate cancer was the most diagnosed cancer among males in groups 1 and 2. Breast cancer was the most common cancer among women in all three categories of countries followed by cervical cancer. However, incidence of prostate, breast, and cervical cancer was not different across the three categories. Lung cancer was the only cancer with significantly higher median incidence recorded in group 3 than in group 1 and group 2 (overall p=0·014).

**Table 1:** Cancer burden by group of fragile states and differences among groups of states

		**Median (25th percentile to 75th percentile)**			**Differences (p value)**		**Worldwide data**
		Group 1	Group 2	Group 3	Overall	Significant differences between groups (p<0·05)	
Incidence (ASR per 100 000 person-years)							
	All cancers	100·80 (95·85–119·00)	116·60 (102·90–137·90)	110·40 (95·70–136·80)	0·33	..	201·0
	Breast cancer	30·60 (25·30–42·85)	28·90 (24·70–41·00)	34·40 (22·00–41·20)	0·80	..	47·8
	Cervix uteri cancer	20·50 (12·85–36·65)	25·10 (20·20–40·80)	14·00 (8·70–22·60)	0·14	..	13·3
	Colon cancer	3·40 (2·65–3·80)	3·30 (2·30–5·10)	4·50 (2·40–5·20)	0·84	..	11·4
	Lung cancer	2·40 (1·95–3·40)	2·90 (2·20–3·90)	9·50 (2·60–12·00)	0·014	2 *vs* 3 and 1 *vs* 3	22·4
	Prostate cancer	32·30 (8·45–37·45)	39·90 (17·10–43·30)	7·50 (3·60–27·40)	0·11	..	30·7
	Rectal cancer	2·90 (2·30–3·30)	2·90 (1·70–3·50)	3·10 (1·80–3·70)	0·96	..	7·6
Estimated prevalent cancer cases diagnosed within the past 5 years per 100 000 population, for all sexes in 2020		90·20 (79·45–109·45)	99·00 (87·40–136·70)	149·20 (115·60–206·20)	0·0053	2 *vs* 3 and 1 *vs* 3	648·5
Mortality (ASR per 100 000 person-years)							
	All cancers	76·50 (72·15–84·95)	90·50 (75·30–103·20)	76·90 (63·20–99·00)	0·33	..	100·7
	Breast cancer	20·00 (15·60–24·70)	17·90 (12·00–24·10)	18·80 (9·60–23·00)	0·41	..	13·6
	Cervix uteri cancer	16·00 (9·90–26·85)	20·20 (14·20–30·80)	8·80 (6·20–16·40)	0·098	2 *vs* 3	7·3
	Colon cancer	2·60 (2·15–3·05)	2·50 (1·80–4·20)	2·90 (1·80–3·30)	0·95	..	5·4
	Lung cancer	2·20 (1·85–3·20)	2·90 (2·10–3·60)	8·80 (2·40–11·10)	0·015	2 *vs* 3 and 1 *vs* 3	18·0
	Prostate cancer	20·40 (5·25–24·40)	23·10 (11·30–26·60)	3·10 (2·00–17·70)	0·073	2 *vs* 3	7·7
	Rectal cancer	2·20 (1·90–2·60)	2·20 (1·20–2·50)	1·90 (1·00–2·40)	0·47	..	3·3
	Total mortality caused by cancer (%)	6·31 (4·52–7·06)	7·37 (6·53–8·91)	11·16 (8·44–12·51)	0·0024	2 *vs* 3 and 1 *vs* 3	17·83
Mortality-to-incidence ratio							
	All cancers	0·76 (0·70–0·78)	0·75 (0·71–0·76)	0·69 (0·67–0·71)	0·0080	2 *vs* 3 and 1 *vs* 3	0·50
	Breast cancer	0·61 (0·56–0·64)	0·58 (0·54–0·62)	0·52 (0·43–0·55)	0·0053	2 *vs* 3 and 1 *vs* 3	0·28
	Cervix uteri cancer	0·77 (0·72–0·78)	0·74 (0·73–0·78)	0·68 (0·63–0·71)	0·0037	2 *vs* 3 and 1 *vs* 3	0·55
	Colon cancer	0·77 (0·75–0·82)	0·77 (0·75–0·80)	0·63 (0·60–0·73)	0·0019	2 *vs* 3 and 1 *vs* 3	0·47
	Lung cancer	0·95 (0·92–0·96)	0·94 (0·93–0·95)	0·92 (0·89–0·92)	0·0021	2 *vs* 3 and 1 *vs* 3	0·80
	Prostate cancer	0·63 (0·60–0·66)	0·63 (0·59–0·64)	0·53 (0·48–0·59)	0·0021	2 *vs* 3 and 1 *vs* 3	0·25
	Rectal cancer	0·81 (0·73–0·85)	0·76 (0·71–0·85)	0·61 (0·56–0·71)	0·0064	2 *vs* 3 and 1 *vs* 3	0·43
DALYs							
	DALYs per 100 000 population, 2019	1557·04 (1250·61–1755·43)	1509·66 (1334·98–2080·36)	1946·30 (1492·50–2797·47)	0·074	1 *vs* 3	3249·00
	DALYs caused by cancer (percentage of total DALYs), 2019	3·31 (2·16–3·51)	3·75 (3·22–4·65)	6·66 (4·43–7·59)	0·0039	2 *vs* 3 and 1 *vs* 3	9·93

Group 1 includes countries with an APC of FSI of 0·2% or higher. Group 1 includes Yemen, Eritrea, Central African Republic, Guinea Bissau, Cameroon, Niger, Nigeria, Burundi, and South Sudan. Group 2 includes countries with an APC between 0·2% and −0·2%. Group 2 includes Afghanistan, Democratic Republic of the Congo, Ethiopia, Somalia, Kenya, Congo (Brazzaville), Uganda, Haiti, Chad, Guinea, and Liberia. Group 3 includes countries with an APC of −0·2% or lower. Group 3 includes Sudan, Myanmar, North Korea, Pakistan, Sierra Leone, Iraq, Nepal, Bangladesh, Zimbabwe, Timor Leste, and Côte d'Ivoire. APC=annual percent change. ASR=age-standardised rate. DALY=disability-adjusted life-years. FSI=Fragile State Index.

Highest median ASR of cancer mortality was observed in group 2 (p=0·33). Cancers with highest median mortality rates were prostate and breast cancers in group 1, prostate and cervical cancers in group 2, and breast cancer, cervical cancer, and lung cancer in group 3. Mortality differences followed a similar pattern to incidence, lung cancer being the only tumour site showing a significantly higher rate in group 3 than in groups 1 and 2 (overall p=0·015). Mortality caused by cancer as a proportion of all-cause mortality was inversely related to the FSI, with group 3 (11·16%) being significantly higher than group 1 (6·31%) and group 2 (7·37%). The M:I ratio in the group 3 countries was significantly lower than in the other two groups for all cancers as well as for the common cancers. Median DALYs caused by cancer increased with the reduction in FSI, being statistically different overall (p=0·0039) and when comparing group 3 to group 1 (p=0·0044) and group 2 (p=0·011).

Regarding cancer risk factors, proportion of the population exposed to outdoor air pollution measured as population-weighted annual averages of exposure to PM_2·5_ was lower in countries in group 1 or 2 than in group 3 (table 2). Exposure to household air pollution, measured as a percentage of population exposed to household air pollution from burning solid fuels for heating and cooking, was significantly higher in groups 1 and 2 (being more than 94% of the population exposed; overall p=0·045). Alcohol consumption documented as litres of pure alcohol consumed per capita was highest in group 1. Median prevalence of obesity ranged from 3·60% to 4·35% among adult men, and from 9·30% to 12·00% among adult women, showing no significant differences among the groups of countries.

**Table 2:** Risk factors and PAF by group of fragile states and differences among groups of states

	**Median (25th percentile to 75th percentile)**			**Differences (p value)**		**Worldwide data**
	Group 1	Group 2	Group 3	Overall	Significant differences between groups (p<0·05)	
**Air pollution**						
Exposure to outdoor air pollution population-weighted annual averages (μg/m^3^) of particulate matter less than or equal to 2·5 μm in aerodynamic diameter at the national level in 2019	46·40 (40·85–67·45)	35·90 (30·40–52·40)	51·10 (29·40–62·60)	0·22	..	21·2 (median)
Percentage of the population exposed to household air pollution from burning solid fuels for heating and cooking in 2019	97·70 (70·20–99·10)	94·90 (86·70–97·50)	75·90 (53·00–82·10)	0·045	2 *vs* 3	11·7 (median)
Total per-capita (aged 15 years or older) consumption of pure alcohol in litres (95% CI) for all sexes, 2016–18	3·90 (0·88–6·83)	2·40 (1·10–6·10)	2·20 (0·40–4·70)	0·65	..	6
**Infections**						
Children aged 1 year covered by hepatitis B immunisation with the third dose of hepatitis B vaccine, 2019 (%)	73·00 (53·00–88·50)	66·00 (50·00–79·00)	90·00 (84·00–95·00)	0·0050	2 *vs* 3 and 1 *vs* 3	83 (2020)
Estimated adult (aged 15–49 years) HIV prevalence for all sexes, 2018 (%)	1·50 (0·50–3·55)	1·30 (0·63–2·15)	0·50 (0·10–2·33)	0·57	..	0·7 (0·6–0·9; 2020)
Human papillomavirus vaccination in national immunisation programme (number of countries that have HPV vaccination programmes per total countries), 2020	1/9	4/11	3/11	..	..	107/194
**Obesity**						
Age-standardised prevalence estimate (%) of obesity (BMI ≥30) among adult men, 2016	4·15 (2·20–5·83)	3·60 (2·80–5·50)	4·35 (2·68–6·03)	0·55	..	17·1 (median)
Age-standardised prevalence estimate (%) of obesity (BMI ≥30) among adult women, 2016	12·00 (8·63–15·73)	11·10 (8·60–13·50)	9·30 (5·30–17·73)	0·74	..	22·7 (median)
**Smoking**						
Number of fully implemented MPOWER measures, 2018	1 (0–1)	1 (0–2)	0 (0–1)	0·51	..	82/195 countries with ≥2 MPOWER measures fully implemented
Current use of any tobacco product (ASR per 100) for all sexes (aged 15 years or older), 2018	8·95 (6·63–14·68)	9·75 (7·30–11·80)	21·10 (17·54–38·48)	0·0015	2 *vs* 3 and 1 *vs* 3	22·05 (median)
Prevalence (%) of daily smoking for men	11·60 (9·95–13·55)	14·10 (9·80–21·40)	24·80 (20·50–37·30)	0·0055	2 *vs* 3 and 1 *vs* 3	21·50 (median)
Prevalence (%) of daily smoking for women	0·90 (0·55–1·55)	1·40 (1·00–1·80)	2·00 (0·90–7·30)	0·15	..	3·85 (median)
**PAF (%)**						
Cancer deaths attributable to alcohol use (by alcohol drinking in men aged 15 years and older), 2017	4·56 (3·24–8·29)	5·42 (4·68–7·58)	3·87 (1·49–5·46)	0·28	..	4–5 (2016)
Cancer deaths attributable by chewing tobacco, 2017	0·16 (0·11–0·45)	0·14 (0·09–0·27)	0·51 (0·10–3·96)	0·15	..	0·07 (median)
Cancer deaths attributable by tobacco, 2017	8·36 (4·83–10·61)	8·26 (6·31–8·97)	20·00 (12·42–23·70)	0·0050	2 *vs* 3 and 1 *vs* 3	25·0
Cancer cases attributable to infections, 2018	22·40 (17·25–31·20)	21·20 (17·20–34·80)	18·80 (12·00–24·70)	0·21	..	13·0
Cancer cases attributable to obesity, 2012	0·93 (0·61–1·40)	0·68 (0·54–1·10)	1·10 (0·72–1·70)	0·29	..	3–4
Cancer deaths attributable to occupational hazard, 2017	0·80 (0·57–0·95)	0·73 (0·65–1·05)	1·71 (0·81–1·94)	0·015	2 *vs* 3 and 1 *vs* 3	2–8

Group 1 includes countries with an APC of FSI of 0·2% or higher. Group 1 includes Yemen, Eritrea, Central African Republic, Guinea Bissau, Cameroon, Niger, Nigeria, Burundi, and South Sudan. Group 2 includes countries with an APC between 0·2% and −0·2%. Group 2 includes Afghanistan, Democratic Republic of the Congo, Ethiopia, Somalia, Kenya, Congo (Brazzaville), Uganda, Haiti, Chad, Guinea, and Liberia. Group 3 includes countries with an APC of −0·2% or lower. Group 3 includes Sudan, Myanmar, North Korea, Pakistan, Sierra Leone, Iraq, Nepal, Bangladesh, Zimbabwe, Timor Leste, and Côte d'Ivoire. APC=annual percent change. ASR=age-standardised rate. FSI=Fragile State Index. PAF=population-attributable fraction.

Current use of any tobacco product increased when moving from group 1 to 3 (p=0·0015), being statistically higher in group 3 (21·1 smokers per 100) than in group 1 (p=0·0067) and group 2 (p=0·0012). A similar pattern could be seen in prevalence of daily smoking among men (ranging from 11·60% to 24·80%), but not among women, with 2·00% or less being daily smokers. Group 1 and 2 countries had a median of one fully implemented MPOWER measure for tobacco control in place, whereas group 3 had a median of zero measures.

The proportion of cancer deaths attributable (PAF) to tobacco was inversely related to the FSI (p=0·0050) and was higher in group 3 than group 1 (p=0·0071) and group 2 (p=0·0034). Cancer deaths attributable to occupational exposures were also different overall (p=0·015) and higher in group 3 than in groups 1 and 2.

Infections were responsible for 22·40% of cancer cases in groups 1 and 21·20% in group 2, whereas they were responsible for 18·80% in group 3, with no statistical difference. A significantly lower coverage of the third dose of the hepatitis B vaccine given to children aged 1 year was documented in countries of group 2 than in group 1 and group 3. There was no difference in the estimated HIV prevalence among people aged 15–49 years, median prevalence ranging from 0·50% in group 3 to 1·50% in group 1. Only eight countries had fully or partially introduced human papillomavirus (HPV) vaccination in their immunisation programme.

Most countries (25 [81%] of 31) have an action plan that integrates several non-communicable diseases and their risk factors, being multisectoral in all cases except one (table 3). 15 countries had an updated cancer plan, with the highest proportion being among countries in group 3. 15 countries had updated cancer-management guidelines, but only six used the guidelines in at least 50% of their facilities. 12 countries reported having a cervical cancer early-detection programme or guidelines, all of them being from group 2 (five [45%] of 11) and group 3 (seven [64%] of 11). An imPACT review mission had been done in more than half of the countries (21 [68%] of 31).

**Table 3:** Political commitment to improving cancer control by group of countries

	**N/total countries**			**Worldwide data**
	Group 1	Group 2	Group 3	
**Documented plans and guidelines**				
Updated non-communicable diseases integrated plan	7/9	8/11	10/11	172/194
Updated multisectoral non-communicable diseases integrated plan	6/7	8/8	10/10	168/172
Cancer plan (updated)	3/9	5/11	7/11	135/192
Cancer management guidelines (updated)	1/9	7/11	7/11	135/193
Cancer management guidelines used in 50% of facilities	0/1	2/7	4/7	92/135
Cervical cancer early detection programme and guidelines	0/9	5/11	7/11	121/194
**External evaluation**				
imPACT review mission	5/9	8/11	8/11	90/194

Group 1 includes countries with an APC of FSI of 0·2% or higher. Group 1 includes Yemen, Eritrea, Central African Republic, Guinea Bissau, Cameroon, Niger, Nigeria, Burundi, and South Sudan. Group 2 includes countries with an APC between 0·2% and −0·2%. Group 2 includes Afghanistan, Democratic Republic of the Congo, Ethiopia, Somalia, Kenya, Congo (Brazzaville), Uganda, Haiti, Chad, Guinea, and Liberia. Group 3 includes countries with an APC of −0·2% or lower. Group 3 includes Sudan, Myanmar, North Korea, Pakistan, Sierra Leone, Iraq, Nepal, Bangladesh, Zimbabwe, Timor Leste, and Côte d'Ivoire. APC=annual percent change. FSI=Fragile State Index. imPACT=integrated mission of Prograame of Action for Cancer Therapy.

Regarding health system financing (table 4), there were overall differences in current health expenditure per capita (ranging from US$41·83 to $66·03; p=0·021) and domestic general government health expenditure per capita ($4·29 to $14·86; p=0·0039), which were significantly higher in group 3 when comparing with group 1 (p=0·013 and p=0·0025, respectively) and group 2 (p=0·023 and p=0·013, respectively). Moreover, out-of-pocket expenditure was 48·72% in group 1, 42·68% in group 2, and 51·07% in group 3 of current health expenditure (p=0·888), and out-of-pocket expenditure per capita was statistically higher in group 3 ($32·57) than in group 2 ($19·93; p=0·016).

**Table 4:** Health system financing, infrastructure, and workforce by group of fragile states and differences among groups of states

	**Median (25th percentile to 75th percentile)**			**Differences (p value)**		**Worldwide data**
	Group 1	Group 2	Group 3	Overall	Significant differences between groups (p<0·05)	
**Financing**						
CHE as a percentage of gross domestic product, 2018	6·70 (3·94–7·64)	4·63 (3·30–6·98)	4·42 (3·88–5·05)	0·53	..	6·33 (median)
CHE per capita in US$, 2018	41·83 (24·72–54·02)	44·28 (27·99–53·44)	66·03 (54·10–105·35)	0·021	2 *vs* 3 and 1 *vs* 3	389·87 (median)
GGHE-D as a percentage of CHE, 2018	12·82 (6·99–22·32)	16·72 (14·30–28·06)	26·51 (16·44–38·68)	0·074	1 *vs* 3	54·05 (median)
GGHE-D per capita in US$, 2018	4·29 (3·26–9·04)	6·56 (4·43–12·94)	14·86 (8·67–43·64)	0·0039	2 *vs* 3 and 1 *vs* 3	204·50 (median)
OOP expenditure as a percentage of CHE, 2018	48·72 (29·62–75·30)	42·68 (37·65–60·94)	51·07 (35·66–68·13)	0·89	..	29·33 (median)
OOP per capita in US$, 2018	18·59 (7·51–40·61)	19·93 (14·57–25·64)	32·57 (27·28–41·20)	0·090	2 *vs* 3	93·89 (median)
**Infrastructure**						
Number of radiotherapy machines per 10 000 patients with cancer, 2019	0·10 (0·00–0·75)	0·00 (0·00–0·15)	2·70 (0·00–3·30)	0·019	2 *vs* 3 and 1 *vs* 3	6·5 (median)
Number of mammographs per 10 000 patients with cancer, 2020	9·50 (2·68–16·27)	8·28 (2·12–17·53)	6·90 (0·81–8·86)	0·48	..	24·5 (median)
Number of radiotherapy units (LINAC and Cobalt), 2020	0 (0·00–3·00)	1·00 (0·00–2·00)	6·00 (2·25–9·00)	0·031	2 *vs* 3 and 1 *vs* 3	2 (median)
Pathology services generally available in the public sector (number of countries per total countries), 2019	5/9	4/11	5/11	..	..	157/194
Palliative care (community and home-based care) generally available (number of countries per total countries), 2019	0/9	0/11	2/10	..	..	78/194
Consumption of opioids in S-DDD* for pain management, 2015–17	0 (0–0)	2 (0–25)	9 (2–21)	0·0078	1 *vs* 2 and 1 *vs* 3	69 (median)
Number of dedicated public cancer centres per 10 000 patients with cancer, 2019	0·00 (0·00–1·05)	0·47 (0·04–15·76)	0·96 (0·68–7·17)	0·17	..	2 (median)
Number of dedicated private cancer centres per 10 000 patients with cancer, 2019	0·00 (0·00–0·16)	0·00 (0·00–1·25)	0·06 (0·00–0·58)	0·37	..	0 (median)
**Workforce**						
Number of radiation oncologists per 10 000 patients with cancer, 2019	0·00 (0·00–6·04)	0·00 (0·00–0·00)	1·78 (1·72–3·59)	0·25	..	14 (median)
Number of medical physicists per 10 000 patients with cancer, 2019	3·87 (2·52–7·61)	0·00 (0·00–0·71)	7·92 (3·95–22·34)	0·0061	1 *vs* 2 and 2 *vs* 3	12 (median)
Number of surgeons per 10 000 patients with cancer, 2019	44·19 (29·77–98·56)	51·65 (19·56–73·95)	106·76 (62·99–184·96)	0·059	2 *vs* 3	319 (median)

Group 1 includes countries with an APC of FSI of 0·2% or higher. Group 1 includes Yemen, Eritrea, Central African Republic, Guinea Bissau, Cameroon, Niger, Nigeria, Burundi, and South Sudan. Group 2 includes countries with an APC between 0·2% and −0·2%. Group 2 includes Afghanistan, Democratic Republic of the Congo, Ethiopia, Somalia, Kenya, Congo (Brazzaville), Uganda, Haiti, Chad, Guinea, and Liberia. Group 3 includes countries with an APC of −0·2% or lower. Group 3 includes Sudan, Myanmar, North Korea, Pakistan, Sierra Leone, Iraq, Nepal, Bangladesh, Zimbabwe, Timor Leste, and Côte d'Ivoire. APC=annual percent change. FSI=Fragile State Index. CHE=current health expenditure. GGHE-D=domestic general government health expenditure. LINAC=linear accelerator. OOP=out-of-pocket expenditure. S-DDD=defined daily doses for statistical purposes.

* Codeine, dextropropoxyphene, dihydrocodeine, fentanyl, hydrocodone, hydromorphone, ketobemidone, morphine, oxycodone, pethidine, tilidine, and trimeperidine.

As for infrastructure, number of radiotherapy machines (p=0·019) and radiotherapy units (p=0·031) available per 10 000 patients with cancer differed overall, and between groups 1 and 3, and groups 2 and 3. There was no difference in number of mammographs, with less than 10 per 10 000 patients with cancer in all groups. Pathology services were generally available in the public sector in only 14 countries, whereas only two countries from group 3 had access to palliative care. The consumption of opioids for pain management was different among the three groups (p=0·0078) and when comparing group 1 (with no consumption) with groups 2 and 3 (median two defined daily doses for group 2 and nine defined daily doses for group 3; table 4). Moreover, the number of dedicated public cancer centres and dedicated private centres per 10 000 patients with cancer was less than one in the three groups.

The number of medical physicists per 10 000 patients with cancer was the only variable related to workforce that was different between groups 1 and 2, being statistically higher in the most fragile states (3·87; p=0·013). The number of surgeons per 10 000 patients with cancer in group 3 (106·76) was nearly two times that of group 2 (51·65), the difference being statistically significant (p=0·022).

## Discussion

Fragile states have an ageing population and will start or are already undergoing an epidemiological transition from communicable diseases to non-communicable diseases. Fragile states face a relatively low ASR of cancer incidence, lower than the worldwide rate for all cancers (201·0 per 100 000 person-years), and common cancers such as breast (47·8 per 100 000 person-years) and lung (22·4 per 100 000 person-years) cancers. However, cervical cancer incidence is higher than the global ASR (13·3 per 100 000 person-years), as is prostate cancer in groups 1 and 2 (worldwide ASR 30·7 per 100 000 person-years).^7^ Cancer burden is expected to rise and there will be a change in the predominant cancers caused by a change in the risk factors.

Although the majority of cancers associated with infections are highly preventable, these cancers contribute to 20% of cancer cases in the fragile states, which is higher than the world proportion of 13·0%.^12^ Despite the high burden of cervical cancer in the fragile countries, less than 20% have been able to introduce HPV vaccination in their national immunisation programme, even though more than 75% of countries are eligible for support from Gavi, The Vaccine Alliance. Coverage of cervical cancer screening is either low or unknown in these countries. Low vaccination and screening rates raise concerns about the feasibility of achieving the WHO 2030 targets for cervical cancer elimination. Furthermore, ensuring high coverage to hepatitis B vaccination starting with an at-birth dose and improving access to treatment of hepatitis C would significantly reduce liver cancer burden.

Tobacco consumption is an emerging risk factor. Between 1980 and 2016 there was a 55% increase in annual cigarette consumption in the WHO African region, where most of the fragile states are situated.^18^ Fragile states are significantly lagging in effective implementation of the Framework Convention on Tobacco Control recommendations. All the MPOWER measures need to be strengthened.

The approaches to control pollution and exposure to environmental carcinogens might be more complex and require a multisectoral approach with a strong legislative framework. The population of fragile states is exposed to outdoor air pollution concentrations far higher than the WHO Air Quality Guideline of 10 μg/m^3^, and in 29 of 31 countries more than 50% of the population uses solid fuels. Although several countries have reduced their household air pollution in the period of 2010–19, others such as Ethiopia and Nigeria have increased pollution. This increase could be caused by population growth outpacing the reduction in use of solid fuels.^13^

Governments show some level of commitment to cancer control through doing imPACT review missions, but cancer control planning is weak in fragile states, with only 15 countries having an updated cancer control plan, although the status of their implementation is unknown. Moreover, to develop cancer control policies tailored to the local context, countries need to monitor disease burden through high-quality cancer registries. Less than half of the countries have population-based cancer registries that collect information in a standardised manner and allow cancer burden assessment, with high-quality data being frequently unavailable.^26^ Challenges faced by fragile states can be an absence of legislation mandating cancer registration, disruption of cancer registration because of political instability, weak health infrastructure, scarcity of trained professionals, and scarcity of funding. Conflicts and natural disasters can cause a migration of population to neighbouring countries, in which cancer services can be a neglected dimension of displaced people's health and they may not even collect information on the non-national population with cancer.^27^ Fragile states need to address these issues to initiate or improve cancer registration and have more reliable population cancer statistics to better plan and implement cancer control, which the Global Initiative for Cancer Registry development can support.

The fact that fragile states are not prepared for cancer control is also suggested by the high M:I ratio (groups 1 and 2 in particular), which is attributed to a late stage at diagnosis and limited access to good-quality treatment. An early-diagnosis approach can substantially reduce delays in diagnostic and treatment pathways and improve survival and should be prioritised in all fragile states. Screening for cervical cancer in primary-care settings using a screen-and-treat approach needs to be implemented in fragile states with high cervical cancer burden. WHO best buys can provide guidance on interventions for primary and secondary cancer prevention that are resource stratified.

Contextually appropriate and evidence-based treatment of cancers should be promoted. Oncology infrastructure and the workforce in fragile states are far from being adequate. Access to radiation therapy estimated in 14 of the 31 studied fragile states ranged from 2·4% in Ethiopia to 35·4% in Sudan.^28^ In Africa, even though there was a 45% increase in radiotherapy capacity between 2012 and 2020, coverage has only increased 2·7%. Some countries have even decreased their capacity to deliver radiotherapy, which raises concerns about equipment maintenance and trained workforces.^29^ Limited access to treatment facilities is associated with advanced stage at treatment, inappropriate treatment, a worse prognosis, worse quality of life, and an economic burden, including loss of earnings or loss of productivity for patients with cancer and their caregivers.^30^ Enhancing local capacities so that countries are able to perform high-quality oncosurgery and administer inexpensive chemotherapy can, to some extent, compensate for limited access to radiation therapy.

Although cancer explains the largest proportion of the burden of serious health-related suffering,^31^ only people living in two fragile states have access to palliative care. A survey collected data from 81 countries on end-of-life health system performance, including palliative care, among them 12 fragile states represented in this manuscript, and graded their score on the quality of end-of-life care on a scale from A to F, with being A the best. Of the 12 fragile states, seven obtained the lowest grade.^32^

The strength of this Article is the inclusion of information from many data sources, allowing a comprehensive analysis of cancer burden and control in the fragile states. However, we used sources from the UN and global institutions, aiming to have official and global information from health authorities in the countries studied, this approach has limitations, and in some cases, information was not available for all fragile states. This scarcity of data refers not only to incidence and mortality,^26^ but also to cancer outcomes.^33^ Additionally, use of the M:I ratio as a proxy for survival is not valid for all cancers sites.^34^ Moreover, use of cross-sectional data hinders the examination of trends over time of cancer burden, cancer risk factors, and cancer control measures in place. Future research should analyse these trends and assess their relationship with specific dimensions of the FSI.

Although several recommendations have been given on how to improve cancer control, fragile states need to have a quality action plan. WHO offers guidance on this process, which will enable countries to assess their health system readiness and develop a plan on the interventions to improve quality in cancer control.^35^ Countries need to have a plan with realistic priorities, a sustained financing mechanism for implementation, and systematic monitoring and evaluation of the implementation process.^36^ Alternative funding mechanisms should be explored, such as public–private partnerships or procurement mechanisms that allow individual countries to participate in collective, multicountry negotiations to secure access to essential services.^37^

In summary, fragile states are facing an ageing population and an epidemiological transition, but are not adequately tackling cancer risk factors or planning to face future challenges. Although there is some level of commitment of governments to cancer control, fragile states need to develop documented cancer plans and guidelines with an allocated budget for implementation. This planning should include an adequate infrastructure and workforce to provide early diagnosis and adequate treatment to patients with cancer. This is a long path, but countries with a decreasing fragility trend are progressing with their cancer control measures, aligned with the UN target goal of reducing premature mortality from non-communicable diseases.

## Data sharing

All data are publicly available, except for the updated multisectoral non-communicable disease integrated plans, updated cancer plans, cancer management guidelines used in 50% of facilities, and number of dedicated private centres per 10 000 patients with cancer, which can be shared on request to the corresponding author.

## Declaration of interests

We declare no competing interests.

## Supplementary Materials

Arabic translation of the abstract

French translation of the abstract
