# Supplementary material for: Cancer burden and status of cancer control measures in fragile states: a comparative analysis of 31 countries
Source: Lancet Glob Health. 2022 Sep 13;10(10):e1443–52. doi: 10.1016/S2214-109X(22)00331-X (PMC9638035; doi:10.1016/S2214-109X(22)00331-X)
Supplement: Arabic translation of the abstract [file mmc1.pdf]

THE LANCET
Global Health

Supplementary appendix 1

This translation in Arabic was submitted by the authors and we reproduce it as supplied. It has not been peer reviewed. *The Lancet*'s editorial processes have only been applied to the original in English, which should serve as reference for this manuscript.

تم تقديم هذه الترجمة باللغة العربية من قبل المؤلفين ونعيد إنتاجها كما هو مُقدم. إنها لم تخضع لاستعراض الأقران. تم تطبيق عمليات تحرير لانسيت فقط على النص الأصلي باللغة الإنجليزية، والذي يجب أن يكون بمثابة مرجع لهذه المخطوطة.

Supplement to: Mosquera I, Ilbawi A, Muwonge R, Basu P, Carvalho AL. Cancer burden and status of cancer control measures in fragile states: a comparative analysis of 31 countries. *Lancet Glob Health* 2022; **10:** e1443–52.

عبء السرطان و تدابير مكافحته في الدول الهشة : تحليل مقارن 31 دولة

موجز

خلفية إن المعطيات المنشورة عن إحصائيات السرطان والسياسات المتداولة لمكافحته تبقى محدودة في الدول الهشة. يصف هذا العمل عبء السرطان فى هذه الدول و التدابير اللازمة لمكافحته

منهجية في هذا التحليل المقارن ، تم اختيار الدول الهشة التي تقدم درجة مؤشر الدول الهشة 90 درجة أو أكثر) للضعف (لمدة 10 سنوات على الأقل خلال الفترة 2006ء2020. تم اختيار الدول التي لديها بيانات أقل من 10 سنوات إذا كانوا في حالة تأهب للهشاشة خلال جميع السنوات. تم جمع معلومات عن عبء السرطان ، وانتشار عوامل خطر الإصابة بالسرطان ، والجزء الذي يعزى إلى السكان ، والالتزام السياسي ، والتمويل الصحي ، وقدرة النظام الصحي. تم حساب معدل الإصابة بالسرطان والوفيات على أساس البيانات المأخوذة من سجلات السرطان القائمة على السكان ، المقدرة بالنمذجة التي تستخدم نسب الوفيات إلى الحدوث ونسب الإصابة إلى الوفيات المشتقة من سجلات السرطان في البلدان المجاورة ، أو متوسط المعدلات في بلدان مجاورة مختارة. للمقارنة الإحصائية ، الدول الهشة تم تجميعها على أساس النسبة المئوية للتغير السنوي لمؤشر الدول الهشة ، مع إظهار المجموعة 1 اتجاهًا متزايدًا للهشاشة) 0 2٪ أو مرتفع لها (المجموعة 2 اتجاه هشاشة مستقر نسبيًا بين(0 2٪ و –0 2٪) (والمجموعة 3 اتجاه هشاشة متناقص (من –0 2٪ أو أق ل

نتائج بشكل عام ،كان عبء السرطان المقدر في 31 دولة هشة مختارة أقل من المعدلات العالمية ، باستثناء سرطان عنق الرحم وسرطان البروستاتا. نسبت حالات السرطان إلى العدوى(22-40٪ في المجموعة 1 ، 21- 20٪ في المجموعة 2 ، و 18- 80٪ في المجموعة 3) بنسبة أعلى في الدول الهشة منها على الصعيد العالمي (13 -0٪). أظهرت المجموعتان 1 و 2 تعرضًا أعلى بكثير لتلوث الهواء المنزلي (97-70٪ في المجموعة 1 و 94 90٪ في المجموعة 2) ، بينما زاد استخدام التبغ الحالي لدى الرجال من المجموعة 1 إلى المجموعة 3 ، مع حدوث سرطان الرئة وكانت الوفيات أعلى في المجموعة 3. ومع ذلك ، نفذ 25 دولة واحدة فقط أو لم تنفذ أي تدابير لمكافحة التبغ. أظهرت البلدان إنفاقًا شخصيًا قدره 48 72٪ في المجموعة 1 ، و 42 68٪ في المجموعة 2 ، و 51 07٪ في المجموعة 3 ، وكان لدى نصف البلدان فقط خطة محدثة لمكافحة السرطان أو إدارة السرطان القواعد الارشادية

التفسير بدأت الدول الهشة التحول الوبائي لكنها ما زالت لا تنفذ تدابير كافية لمكافحة السرطان. هناك حاجة إلى تطوير خطط وإرشادات موثوقة لمكافحة السرطان ، وإنشاء آليات مالية للتنفيذ.
