# Supplementary material for: Cancer burden and status of cancer control measures in fragile states: a comparative analysis of 31 countries
Source: Lancet Glob Health. 2022 Sep 13;10(10):e1443–52. doi: 10.1016/S2214-109X(22)00331-X (PMC9638035; doi:10.1016/S2214-109X(22)00331-X)
Supplement: French translation of the abstract [file mmc2.pdf]

THE LANCET
Global Health

Supplementary appendix 2

This translation in French was submitted by the authors and we reproduce it as supplied. It has not been peer reviewed. *The Lancet*'s editorial processes have only been applied to the original in English, which should serve as reference for this manuscript.

Cette traduction en français a été proposée par les auteurs et nous l'avons reproduite telle quelle. Elle n'a pas été examinée par des pairs. Les processus éditoriaux du *Lancet* n'ont été appliqués qu'à l'original en anglais et c'est cette version qui doit servir de référence pour ce manuscrit.

Supplement to: Mosquera I, Ilbawi A, Muwonge R, Basu P, Carvalho AL. Cancer burden and status of cancer control measures in fragile states: a comparative analysis of 31 countries. *Lancet Glob Health* 2022; **10:** e1443–52.

Fardeau du cancer et statut des mesures de lutte contre le cancer dans les États fragiles: une analyse comparée de 31 pays

Résumé

Contexte Les informations sur les statistiques du cancer et les politiques de lutte contre le cancer sont limitées dans les États fragiles. Cet article décrit le fardeau du cancer et le statut des mesures de lutte contre le cancer dans ces pays.

Méthodes Dans cette analyse comparée, les États fragiles présentant un score de 90.0 or plus de Indice des États fragiles (FSI) «d'alerte pour la fragilité» pendant au moins 10 ans entre 2006 et 2020 ont été sélectionnés. Les pays dont les données disponibles étaient inférieures à 10 ans ont été inclus lorsque l'ensemble de la période couverte correspondait à un état «d'alerte pour la fragilité». Des informations sur le fardeau du cancer, la prévalence des facteurs de risque du cancer, la fraction attribuable dans la population (FAP), ainsi que sur l'engagement politique, le financement de la santé et la capacité du système de santé ont été recueillies. L'incidence et la mortalité des cancers ont été obtenus par trois modes: calculées à partir de données provenant de registres du cancer dans la population, estimées à l'aide d'une modélisation utilisant les ratios mortalité-incidence et les ratios incidence-mortalité tirés des registres du cancer des pays voisins ou en utilisant la moyenne des taux de certains pays voisins. À des fins de comparaison statistique, les États fragiles ont été regroupés en fonction de la variation de pourcentage annuelle (APC) du FSI, le groupe 1 présentant une tendance à la hausse de la fragilité (APC 0·2% ou supérieur), le groupe 2 une tendance à la stabilité relative (APC entre 0·2% et –0·2%), et le groupe 3 une tendance à la baisse (APC allant de –0·2% à des taux inferieurs).

Résultats Dans l'ensemble, le fardeau estimé du cancer dans les 31 États fragiles sélectionnés était inférieur aux taux mondiaux, sauf pour le cancer du col de l'utérus et celui de la prostate. La proportion de cas de cancer attribuée à des infections (22·40% dans le groupe 1, 21·20% dans le groupe 2 et 18·80% dans le groupe 3) était plus élevée dans les États fragiles qu'au niveau mondial (13·0%). Tandis que les groupes 1 et 2 présentaient une exposition significativement plus élevée à la pollution atmosphérique domestique (97·70% dans le groupe 1 et 94·90% dans le groupe 2), le tabagisme actuel parmi les hommes augmentait du groupe 1 au groupe 3. Par ailleurs, l'incidence du cancer du poumon et la mortalité étant plus élevées dans le groupe 3. Cependant, 25 pays n'avaient mis en œuvre qu'une seule voire aucune mesure MPOWER pour la lutte antitabac. La dépense directe retrouvée au sein des pays était de 48·72% dans le groupe 1, 42·68% dans le groupe 2 et 51·07% dans le groupe 3. Seule la moitié des pays disposaient d'un plan de lutte contre le cancer ou de protocoles actualisés relatifs à la prise en charge du cancer.

Interprétation Les États fragiles ont entamé une transition épidémiologique mais ne mettent toujours pas en œuvre suffisamment de mesures de lutte contre le cancer. Il est nécessaire d'élaborer des plans et des protocoles de lutte contre le cancer fiables et de créer des mécanismes financiers pour leur application.
