# Supplementary material for: Cancer burden and status of cancer control measures in fragile states: a comparative analysis of 31 countries
Source: Lancet Glob Health. 2022 Sep 13;10(10):e1443–52. doi: 10.1016/S2214-109X(22)00331-X (PMC9638035; doi:10.1016/S2214-109X(22)00331-X)
Supplement: Supplementary appendix 3 [file mmc3.pdf]

THE LANCET
Global Health

Supplementary appendix 3

This appendix formed part of the original submission and has been peer reviewed. We post it as supplied by the authors.

Supplement to: Mosquera I, Ilbawi A, Muwonge R, Basu P, Carvalho AL. Cancer burden and status of cancer control measures in fragile states: a comparative analysis of 31 countries. *Lancet Glob Health* 2022; **10:** e1443–52.

Supplementary table 1. Sources of data.

	Indicator	Source
**Cancer burden**	Incidence (ASR per 100,000)<br>Prevalence<br>Mortality (ASR per 100,000)	Global Cancer Observatory: Cancer Today, International Agency for Research on Cancer (IARC)[8]
	Mortality due to cancer<br>DALYs	Global Burden of Disease Study, Institute for Health Metrics and Evaluation (IHME)[9]
	Mortality to incidence ratio	Calculated
**Cancer risk factors and Population Attributable Fraction (PAF)**	Air pollution	State of global air, Health Effects Institute[14]
	Alcohol consumption<br>Obesity	The Global Health Observatory, World Health Organization (WHO)[15]
	Hepatitis B immunization	The Global Health Observatory, WHO[15]; Immunization coverage, WHO[16]
	HIV prevalence	HIV Country Intelligence, WHO[17]; The Global Health Observatory, WHO[15]
	HPV vaccination	WHO[18]
	Tobacco consumption<br>MPOWER measures	WHO report on Global Tobacco Epidemic, WHO[12]
	Smoking prevalence	The Tobacco Atlas, American Cancer Society and Vital Strategies[19]
	Cancer deaths attributable by alcohol	WHO Global status report on alcohol and health[20]
	Cancer deaths attributable by chewing tobacco<br>Cancer deaths attributable by tobacco<br>Cancer deaths attributable to occupational hazard	Global Burden of Disease Study, IHME[21]
	Cancer cases attributable to infections	Globocan[22]
	Cancer cases attributable to obesity	Globocan[23]
**Documented plans and guidelines**	Updated Non-Communicable Diseases (NCD) Integrated plan<br>Updated multisectoral NCD integrated plan<br>Cancer plan (updated)<br>Cancer management guidelines (updated)<br>Cancer management guidelines: utilized in 50% facilities<br>Cervical cancer: early detection programme/guidelines	Country Capacity Survey, WHO[10]
**External evaluation**	imPACT review mission	IAEA imPACT reviews[11]
**Financing**	Current Health Expenditure (CHE) as % Gross Domestic Product (GDP)<br>CHE per capita in US$<br>Domestic General Government Health Expenditure (GGHE-D) as % CHE<br>GGHE-D per capita in US$<br>Out of pocket expenditure (OOP) as % CHE<br>OOP per capita in US$	WHO Global Health Expenditure Database, WHO[24]
**Infrastructure**	# radiotherapy machines per 10,000 cancer patients	WHO cancer country profiles, WHO[13]
	# mammographs per 10,000 cancer patients	IAEA Medical imAGIng and Nuclear mEdicine global resources database, IAEA[25]
	# radiotherapy units (LINAC + Cobalt)	IAEA[26]
	Pathology services generally available in public sector (reaching 50% or more patients)<br>Palliative care (community/home-based care) generally available<br># dedicated public cancer centres per 10,000 cancer patients<br># dedicated private cancer centres per 10,000 cancer patients	Country Capacity Survey, WHO[10]
	Consumption of opioids* in S-DDD for pain management	WHO cancer country profiles, WHO[13]
**Workforce**	# radiation oncologist per 10,000 cancer patients<br># medical physicist per 10,000 cancer patients	IAEA Medical imAGIng and Nuclear mEdicine global resources database, IAEA[25]
	# surgeons per 10,000 cancer patients	The Global Health Observatory, WHO[15]

ASR: age-standardized rate; S-DDD: defined daily doses for statistical purposes.
* Codeine, dextropropoxyphene, dihydrocodeine, fentanyl, hydrocodone, hydromorphone, ketobemidone, morphine, oxycodone, pethidine, tilidine and trimeperidine
